# FOXO3a/PI3K/Akt pathway participates in the ROS- induced apoptosis triggered by α-ZEL and β-ZEL

**DOI:** 10.1038/s41598-024-64350-8

**Published:** 2024-06-10

**Authors:** Dominika Ewa Habrowska-Górczyńska, Marta Justyna Kozieł, Kinga Anna Urbanek, Karolina Kowalska, Agnieszka Wanda Piastowska-Ciesielska

**Affiliations:** 1 Department of Cell Culture and Genomic Analysis, Medical University of Lodz, Zeligowskiego 7/9, 90-752 Lodz, Poland; 2 BRaIn Laboratories, Medical University of Lodz, Czechoslowacka 4, 92-216 Lodz, Poland

**Keywords:** Zearalenol, α-zearalenol, β-zearalenol, Prostate cancer, FOXO3a, Akt, Cell signalling, Prostate cancer

## Abstract

Zearalenone (ZEN), an estrogenic mycotoxin, is one of the most common food and feed contaminants. Also, its metabolites α-zearalenol (α-ZEL) and β-zearalenol (β-ZEL) are considered to induce oxidative stress, however its effect in prostate cells is not known yet. Our previous observations showed that forehead box transcription factor 3a (FOXO3a) expression is modified in hormone- sensitive cells in the response to mycotoxins, similar to the phosphoinositide 3-kinase (PI3K)/ protein kinase B (Akt) pathway. Thus, this study evaluated the direct molecular effect of α-ZEL and β-ZEL in a dose of 30 µM in hormone-dependent human prostate cancer (PCa) cells with the focus of the involvement of FOXO3a and PI3K/Akt signaling pathway in that effect. We observed that both active metabolites of ZEN reduced cell viability, induced oxidative stress, cell cycle arrest and apoptosis in PCa cells. Furthermore, we observed that FOXO3a as well as PI3K/Akt signaling pathway participate in ZELs induced toxicity in PCa cells, indicating that this signaling pathway might be a regulator of mycotoxin-induced toxicity generally.

## Introduction

Zearalenone (ZEN), a non- steroidal estrogenic mycotoxins, is one of the best known and one of the most common *Fusarium* mycotoxins worldwide. Its incidence is the highest in cereal products, flour, soybean and beer^1^. ZEN is rapidly absorbed after oral administration and in the result of hydroxylation, two active metabolites of ZEN: α-zearalenol (α-ZEL) and β-zearalenol (β-ZEL), together named ZELs, are formed^2^. The ZELs might be also found directly in the molds extracts but in a very low extend^3^. Acute toxicity of ZEN is very rare, however its chronic exposure and estrogenicity may be a global issue^2^. The full mechanism of ZEN (and its metabolites) toxicity is not completely established. So far, reproductive, carcinogenicity, hemotoxic, genotoxicity and immunotoxicity as well as act as of endocrine disruptors have been confirmed^2^. Numerously studies have already evaluated the effect of ZEN in both in vitro as well as in vivo models, however only a few studies considered evaluation of the ZELs. Generally, ZEN and its metabolites are reported to induce oxidative stress, apoptosis, loss of the mitochondrial potential, modulates the expression of Bax and Bcl-2 proteins and modulates the MAPK signaling pathway^4^, the susceptibility of animals to ZELs seems to be species- dependent: pigs are more sensitive to α-ZEL, whereas chickens, cows and sheep seems to be more vulnerable for β-ZEL^5^. However, no safety regulation has been implemented for ZELs^6^ and its effect on human health has not been fully elucidated. Even for ZEN, the detailed molecular mechanism, after the discovery of its estrogenic potential, has not been resolved in details.

Previous studies showed that both ZEN as well as deoxynivalenol (DON) affects viability and cell cycle regulation in cells^7,8^. Phosphoinositide 3-kinase (PI3K)/protein kinase B (Akt) signaling is one of the most deregulated signaling pathways in PCa. That signaling pathway might regulate by a wide range of cell agent on different levels, directly or indirectly. One of the transcriptions factor regulated by PI3K/Akt is forkhead box O3 (FOXO3a). FOXO3a is involved in regulation of diverse cellular processes: apoptosis, cell cycle progression, DNA damage, proliferation and response to ROS in cells^9^. In PCa FOXO3a is known to be correlated with WNT/β-catenin pathway, estrogen receptor β (ERβ), epithelial to mesenchymal transition (EMT) as well as extracellular signal-regulated kinases (ERK) and mitogen-activated protein kinases (MAPK) signaling pathways^10–12^. There is a large group of natural products that modulates the expression and action of FOXO3a in cells: apigenin^13^, resveratrol^14^, diosmetin^15^ as well as sulforaphane^16^. Also mycotoxins have been reported to modulate the FOXO3a in cells. DON was reported to induce apoptosis in small-intestine cells in pigs via modulation of FOXO3a signalling pathway and ERK1/2^17^. The regulation of apoptosis process as well as mitochondrial toxicity and cell death by FOXO3a and ROS/JNK pathway in DON action was also reported in another study^18^. FOXO3a was also reported to participate in fusaric acid and fumonisin B1-induced apoptosis in human hepatic cells HepG2^19^. Nevertheless, the interplay between FOXO3a and PI3K/Akt signalling pathway in the action of mycotoxins in PCa cells has not been elucidated yet. Thus, we decided to evaluate the interplay of FOXO3a and PI3K/Akt in PCa cell in the response to the two most common and potent ZEN metabolites: α-ZEL and β-ZEL. For this purpose, we generated FOXO3a-PC3 cells in which the PI3K/Akt signalling pathway was blocked with selective chemical inhibitor LY294002. The toxic effect of ZELs was evaluated with cell viability, oxidative stress, apoptosis and cell cycle evaluation.

## Results

### α-ZEL and β-ZEL induce toxicity and upregulate expression of FOXO3a and Akt in PC3 cells

**Figure 1:**
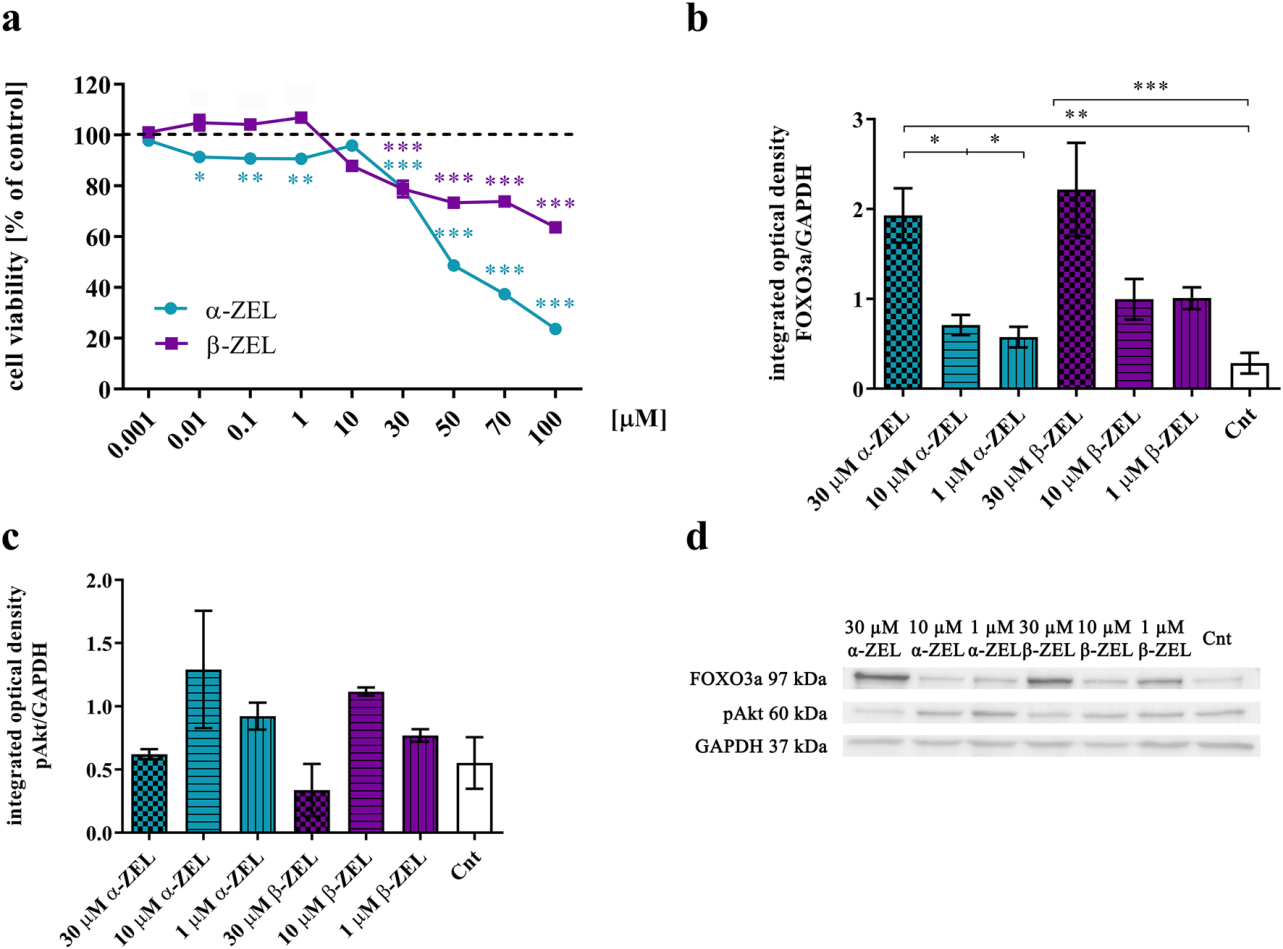
α-ZEL and β-ZEL induce toxicity and upregulate expression of FOXO3a and Akt in PC3 cells. (**a**) Toxicity assay (MTT assay) of α-ZEL and β-ZEL after 24 h incubation in PCa cells. The results are expressed as a % of control (non-treated cells). (**b**) and (**c**) p-Akt and FOXO3a expression after 24 h hours incubation with chosen doses of ZELs evaluated with Western blot. (**d**) representative results of Western blot assay, original blots are presented in Supplementary Figure S1. All the results are expressed as mean ± SE. One-way ANOVA with Bonferroni correction was used as statistical analysis. *p* < 0.05 was considered as significant, *p < 0.05, ** p < 0.001, *** p < 0.001. α-ZEL- α- zearalenol, β-ZEL- β-zearalenol, Cnt- control, non-treated cells.

Firstly, we assessed if α-ZEL and β-ZEL induce cytotoxicity in PC3 cells. After 24 h we observed that both mycotoxins caused a significant decrease in cell viability (Fig. 1a). As expected β-ZEL showed less toxic effect than α-ZEL, in the same concentration range. For the tested dose range IC_50_ value was not reached for β-ZEL, whereas for α-ZEL it accounted 45.19 µM.

Next, we evaluated if tested mycotoxins might affect the expression of FOXO3a and Akt in PC3 cells. For this purpose we chosen a range of concentrations: 30 µM, 10 µM and 1 µM of α/β-ZEL and treated the cells for 24 h. The results showed (Fig. 1b–d) that both α-ZEL and β-ZEL increase the expression of FOXO3a and simultaneously decreased the expression of phospho-Akt. We observed significant increase in the expression of FOXO3a after treatment with 30 µM α-ZEL as compared to control (**p < 0.01) as well as other tested doses of α-ZEL (*p < 0.05). A similar effect was caused by 30 µM of β-ZEL. A not significant decrease in the expression of phospho-Akt was observed for the highest tested dose of mycotoxins, whereas for lower tested doses a slight increase was observed.

### FOXO3a/PI3K/Akt participates in α-ZEL and β-ZEL ROS- induced apoptosis in PCa cells

**Figure 2:**
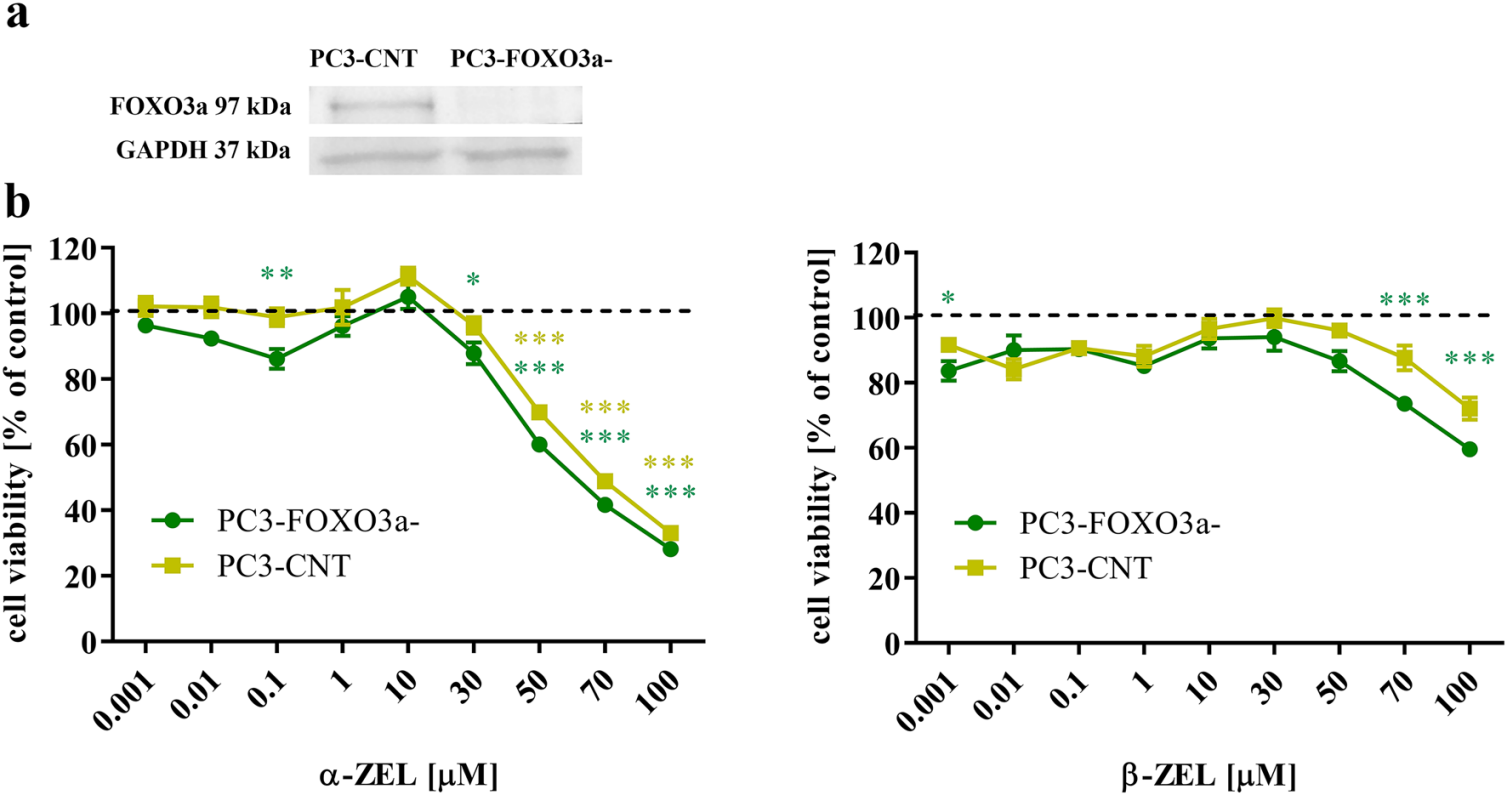
PC3-FOXO3a- cells responded similarly as PC3-CNT cells to α-ZEL and β-ZEL induced cytotoxicity. (**a**) confirmation of silencing of FOXO3a in PC3 cells with Crisp-Cas9 method assessed with Western blot. (**b**) Cell viability curve obtained for generated PC3 cell lines (PC3-FOXO3a- and PC3-CNT) after α-ZEL and β-ZEL treatment for 24 h. One way ANOVA was used for statistical analysis, *p* < 0.05 was considered as statistically significant. *p < 0.05, **p < 0.01, ***p < 0.001. The results are expressed as mean ± SE of 3 replicates. α-ZEL- α- zearalenol, β-ZEL- β-zearalenol.

To confirm the role of FOXO3a in the mycotoxin- induced toxicity, knockdown of FOXO3a was generated with CRISP-Cas9 method. The sufficiency of silencing was confirmed on Western blot and was sufficient for further experiments (Fig. 2a). Next, the cytotoxicity of α-ZEL and β-ZEL was assessed in generated cells. First of all, any significant change in cell viability between PC3-FOXO3a- and PC3-CNT was observed (Fig. 2b). A statistically significant decrease in cells response to α-ZEL was observed in both cell lines as compared to non-treated cells for concentration range 100–30 µM (*p < 0.05, ***p < 0.001) for PC3-FOXO3a- and 100–50 µM (***p < 0.001) for PC3-CNT. The observed statistically significant decrease in cell viability was in the range of 12% to 72% for α-ZEL and 26% to 40% for β-ZEL in PC3-FOXO3a- cells. The statistically significant decrease in cell viability in PC3-CNT was in range of 30% to 67% for α-ZEL. In case of β-ZEL a similar trend was observed, but was significant only for 70 µM and 100 µM (***p < 0.001) for PC3-FOXO3a-. Based on these results the dose of 30 µM of α-ZEL and β-ZEL were chosen for the rest of experiment.

To further understand the role of FOXO3a in ZELs toxicity, the role of FOXO3a and PI3K/Akt was elucidated in oxidative stress, apoptosis and cell cycle regulation. For these experiments the dose of 30 µL of mycotoxin was chosen. The rationale for such a relative high dose of mycotoxins metabolites was based on cell-viability assay, and the fact that aim of this study is to elucidate the molecular mechanism of action of mycotoxins. Both generated cell lines PC3-FOXO3a- and PC3-CNT, were then treated with mycotoxins and/or selective PI3K/Akt inhibitor LY294002.

**Figure 3:**
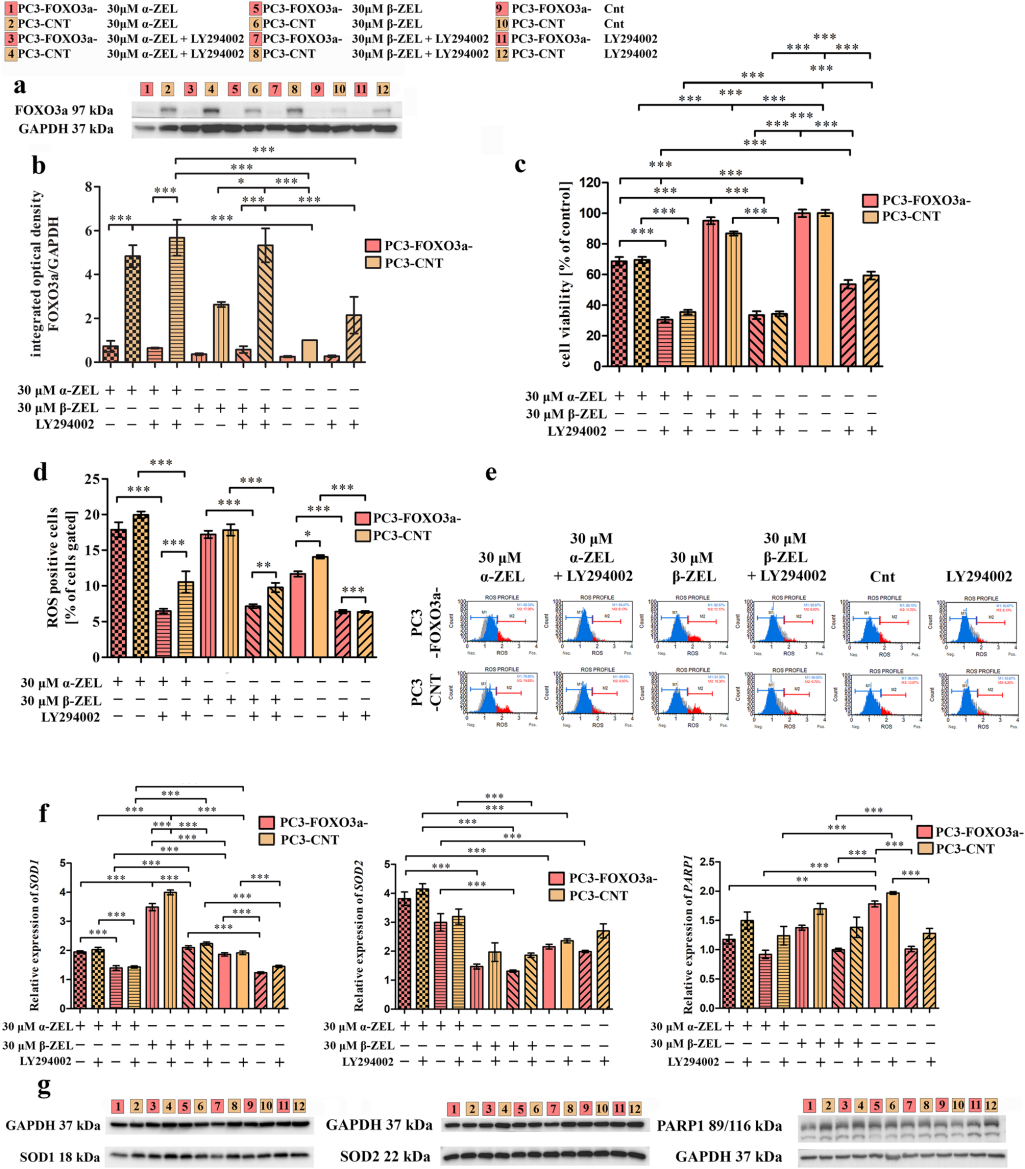
The influence of ZELs on oxidative stress and apoptosis regulation in PC-FOXO3a- and PC3-CNT cells. The modulation of the expression of FOXO3a after ZELs treatment evaluated by Western blot (**a**- representative results of Western blot, **b**- calculate results). Original blots are presented in Supplementary Figure S1. Changes in viability after treatment with mycotoxins and/or selective PI3K/Akt inhibitor LY294002 was evaluated with toxicity assay (MTT assay) after 24 h incubation in PCa cells (**c**). The changes in ROS production in PCa cells was expressed as the percentage of gated cells (**d**). The representative histogram of cells distribution according to ROS (**e**). The relative expression of *SOD1, SOD2* and *PARP1* after ZELs and/or LY294002 treatment (**f**). The relative expression of SOD1, SOD2 and PARP1 evaluated with Western blot (**g**). The values are expressed as the mean ± SE; One-way ANOVA with Bonferroni correction was used for statistical analysis, *p* < 0.05 was considered statistically significant, *** p < 0.001, **p < 0.001, *p < 0.05 as compared to the control. α-ZEL- α- zearalenol, β-ZEL- β-zearalenol, *SOD1*- superoxide dismutase 1, *SOD2*- superoxide dismutase 2, *PARP1*- poly(ADP-ribose) polymerase-1, GAPDH—glyceraldehyde 3-phosphate dehydrogenase, Cnt—control (not treated cells). 1, 2- 30 µM α-ZEL; 3, 4- 30 µM α-ZEL + LY294002; 5, 6- 30 µM β-ZEL; 7, 8- 30 µM β-ZEL + LY294002; 9, 10- Cnt; 11, 12- Cnt + LY294002; PC-FOXO3a- (1; 3; 5; 7; 9; 11), PC3-CNT (2; 4; 6; 8; 10; 12).

Firstly, we evaluated how the silencing of FOXO3a modulated the response of cells to ZELs (Fig. 3a,b). We observed that PC3-CNT cells responded similarly to PC3 cells, where treatment with ZELs resulted in the increased expression of FOXO3a. In all cases addition of LY294002 resulted in the increased expression of FOXO3a as compared to mycotoxin treated- PC3-CNT cells. A similar effect was observed in PC3-CNT cells not treated with mycotoxin. In case of PC3-FOXO3a^−^ cells no changes in the expression of FOXO3a were present, also no changes after addition of LY294002 were observed indicating that FOXO3a might serve as a crucial agent in PI3K/Akt activation.

Next, we move forward and evaluated the cell viability (Fig. 3c). In that case silencing of FOXO3a in cells resulted in no changes in the cell viability as compared to PC3-CNT cells in case of both tested mycotoxins. In all cases, a significant decrease in cells viability was observed after addition of LY294002, also for control cells, confirming the fact, that PI3K/Akt signaling pathway is necessary for cell growth.

Further we conducted ROS (Fig. 3d,e) and apoptosis analysis. We observed that lack of FOXO3a resulted in a slightly decreased number of ROS positive cells, however not significant for ZELs treatment, but significant for control cells (*p < 0.05). Addition of LY294002 resulted in a significant changes between PC3-FOXO3a- and PC3-CNT cells. First of all, a lower extend of ROS positive cells was observed. This fact was possibly caused by the fact that the cells were not dividing, as observed in light microscopy (data not shown). Then, treatment with ZELs and LY294002 resulted in a significant decrease in the number of ROS positive cells in PC3-FOXO3a- as compared to PC3-CNT cells (***p < 0.001 and **p < 0.01 for α-ZEL and β-ZEL, respectively). This effect was contradictory for the one observed for not treated cells (***p < 0.001). To elucidate the oxidative stress induced by ZELs we evaluated the expression of oxidative response enzymes and observed that different superoxide dismutases were activated by α-ZEL and β-ZEL (Fig. 3f,g). β-ZEL significantly induced *SOD1* expression (~ 1.95 fold, ***p < 0.001), whereas α-ZEL caused a significant increase in *SOD2* expression (~ 1.68 fold, ***p < 0.001) as compared to non-treated cells in both cell lines. In all cases PC3-FOXO3a- cells were more sensitive to ZELs, whereas addition of LY294002 resulted in a decreased expression. Similar changes were observed on protein level, however not significant. In case of *PARP1* expression a significant decrease was observed for both ZELs, as well as higher in PC3-FOXO3a- cells as compared to PC3-CNT cells. In all cases addition of LY294002 resulted in a significant decrease in the expression (Table 1.).

**Table. 1:** The integrated optical density of the expression of proteins associated with oxidative stress.

	PC3-FOXO3a^−^			PC3-CNT		
	SOD1	SOD2	PARP1	SOD1	SOD2	PARP1
30 µM α-ZEL	1.3604	1.1017	1.2618	1.4603	1.2028	1.4633
30 µM α-ZEL + LY294002	1.4014	1.1318	1.5290	1.3570	0.9666	1.4558
30 µM β-ZEL	1.5182	0.9478	1.3315	1.4213	0.8816	1.3071
30 µM β-ZEL + LY294002	1.3568	1.0798	1.2422	1.2674	0.9143	1.4112
Cnt	0.8805	0.9927	1.1389	1.0000	1.0000	1.0000
Cnt + LY294002	1.0966	0.9783	1.2426	1.2961	0.8846	1.2309

The results are expressed as a mean. FOXO3a- forkhead box O3, GAPDH- anti-glyceraldehyde 3-phosphate dehydrogenase, SOD1- superoxide dismutase 1, SOD2- superoxide dismutase 2, PARP1- poly (ADP-ribose) polymerase 1, α-ZEL- α- zearalenol, β-ZEL- β-zearalenol, Cnt- control, non-treated cells.

**Figure 4:**
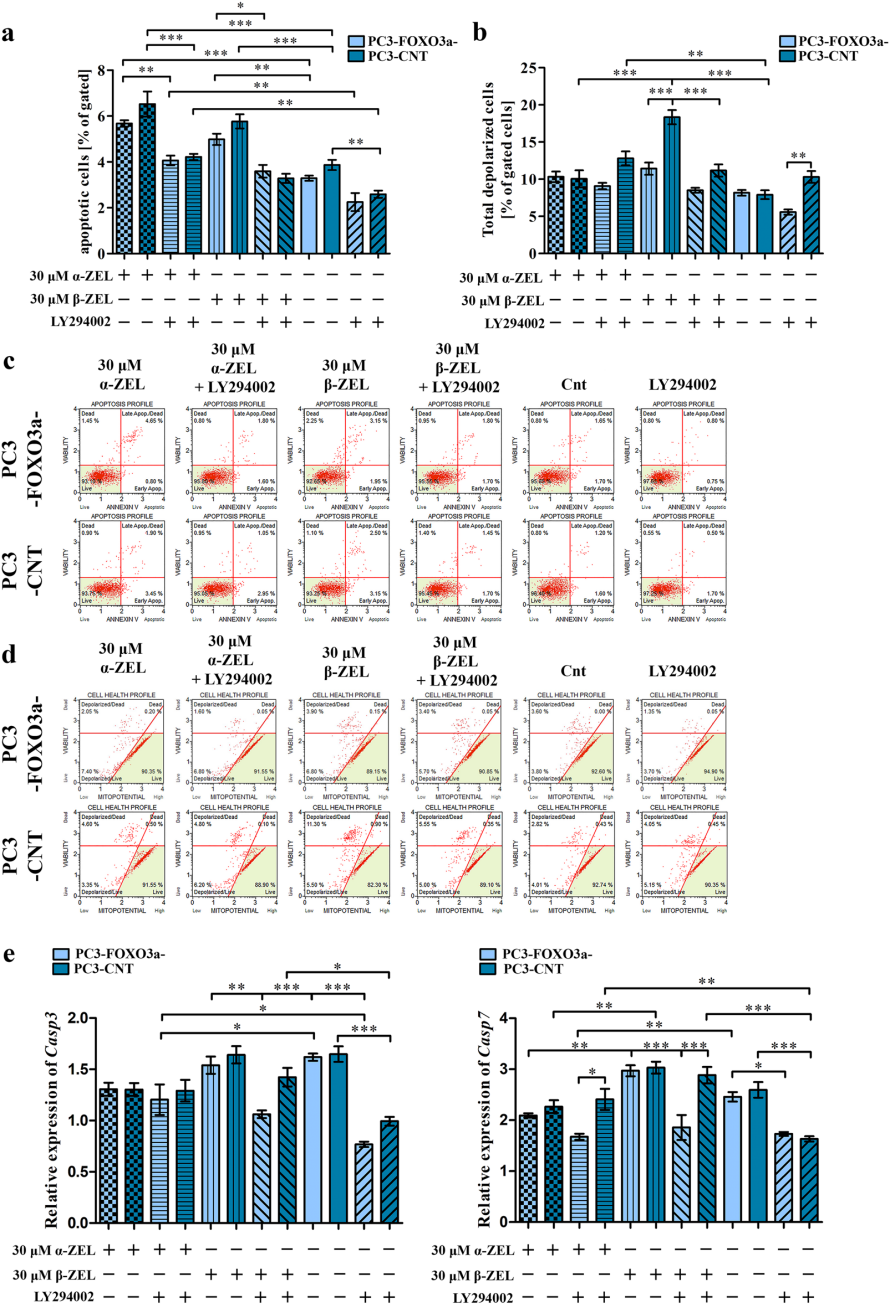
The influence of α-ZEL and β-ZEL on the number of apoptotic cells and mitochondrial potential in PC3-FOXO3a- and PC3-CNT cells. The changes in the number of apoptotic cells (**a**) and mitochondrial potential (**b**) after ZELs and/or LY294002 treatment were measured and expressed as the percentage of gated cells; The representative histogram of apoptosis profile (**c**) and mitochondrial potential (**d**). The relative expression of *Casp3* and *Casp7* after ZELs and/or LY294002 treatment. The values are expressed as the mean ± SE; One- way ANOVA was used for statistical analysis. *p* < 0.05 was considered statistically significant, *** p < 0.001, **p < 0.001, *p < 0.05 as compared to the control. α-ZEL- α- zearalenol, β-ZEL- β-zearalenol, *Casp3*- caspase 3, *Casp7*- caspase 7, Cnt—control (not treated cells).

Then, we decided to evaluate if the increase in the ROS production in cells might be associated with apoptosis in cells. So, firstly we stained the apoptotic cells and counted them with flow cytometry (Fig. 4a,c). We observed that both ZELs significantly increased the number of apoptotic cells (*p < 0.05), and as expected α-ZEL was more toxic than β-ZEL in the same concentration of 30 µM. In all tested conditions PC3-FOXO3a- cells were not significantly less susceptible to apoptosis- inducing condition as compared to PC3-CNT cells, visible also in case of control cells. Blocking of PI3K/Akt resulted in a significant decrease in the number of apoptotic cells in case of both mycotoxins treatment (**p < 0.01, ***p < 0.001) as well as control (**p < 0.01) as compared to cells not treated with LY294002. The observed decrease in the number of apoptotic cells was proportional to the ZELs induced effect.

The observed induction of apoptosis might be associated with changes in the mitochondrial potential, so we decided to elucidate the changes in the mitochondrial potential of cells (Fig. 4b,d). Firstly, we observed that α-ZEL did not significantly increase the number of depolarized cells. Interestingly, a higher increase was observed for β-ZEL, especially in PC3-CNT cells (***p < 0.001). A significant difference was observed between PC3-FOXO3a- and PC3-CNT cells treated with 30 µM β-ZEL (***p < 0.001). When LY294002 was added, a significant decrease in the number of depolarized cells was observed as compared to the same cells treated without LY294002 (***p < 0.001). A contradictory effect was observed for PC3-FOXO3a- cells treated with α-ZEL and control cells PC3-CNT for which addition of LY294002 resulted in the increased number of apoptotic cells.

Next, we evaluated the expression of *Casp3* and *Casp7* (Fig. 4e). Similarly, a different expression regulation was observed after treatment with α-ZEL and β-ZEL. α-ZEL did not change the expression of *Casp7* whereas the expression of *Casp3* was not significantly decreased. Any significant changes were observed between PC3-FOXO3a- and PC3-CNT cells. An insignificant increase in the expression of *Casp7* was observed after treatment of both cell lines with 30 µM of β-ZEL. In case of *Casp3* the expression was almost no changed as compared to control. Addition of LY294002 decreased the expression of both *Casp3* and *Casp7* in a significant manner. In case of mycotoxins treatment the decrease in the expression of *Casp3* was significant in PC3-FOXO3a- cells after β-ZEL treatment as compared to PC3-CNT cells (**p < 0.01). A similar effect was observed for *Casp7* expression both for β-ZEL (***p < 0.001) as well as α-ZEL treatment (*p < 0.05). No such effect was observed for control cells treated with LY294002.

**Figure 5:**
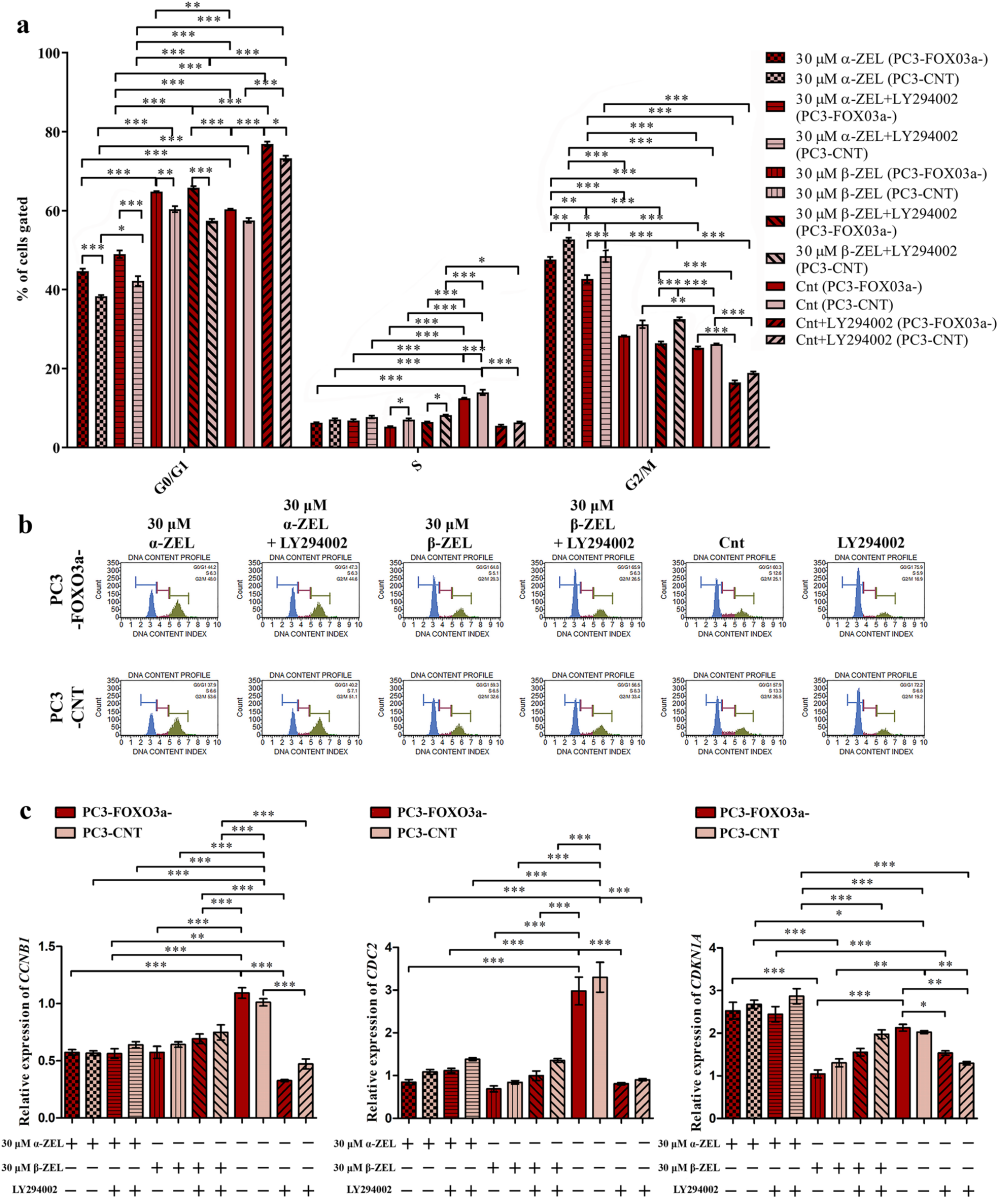
α-ZEL and β-ZEL causes a different modulation of cell cycle progression. The number of PC3-FOXO3a- and PC3-CNT cells in the G0/G1, S and G2/M cell cycle phases (**a**) was expressed as percentage of gated cells. The representative results obtained by flow cytometry with the Cell Cycle Analysis kit (**b**). The relative expression of *CCNB1, CDC2* and *CDKN1A* after ZELs and/or LY294002 treatment (**c**). The results are expressed as the mean ± SE. *p* < 0.05 was considered statistically significant, *** p < 0.001, **p < 0.001, *p < 0.05 as compared to the control. α-ZEL- α- zearalenol, β-ZEL- β-zearalenol, *CCNB1*- cyclin B1, *CDC2*- cyclin-dependent kinase 1, *CDKN1A*- cyclin dependent kinase inhibitor 1A , Cnt—control (not treated cells).

Later, the progression of cell cycle was evaluated (Fig. 5a,b). In case of control cell line a significant decrease in the number of cells in S phase was observed (***p < 0.001). Treatment of α-ZEL and β-ZEL caused a different modulation of cell cycle progression. α-ZEL caused a significant increase in the number of cells in G2/M cell cycle phase as compared to respective control (***p < 0.001) with simultaneous significant decrease in the number of cells in G0/G1 and S cell cycle phase (***p < 0.001). In all treatments with α-ZEL there was a significant decrease in the number of cells in PC3-FOXO3a- cells as compared to PC3-CNT cells in G2/M cell cycle phase with increase in G0/G1 cell cycle phase. Contradictory, β-ZEL caused a significant decrease in the number of cells in S and G2/M cell cycle phase (***p < 0.001) and increased the number of cells in G0/G1 cell cycle phase (***p < 0.001). Addition of LY294002 in case of control cells significantly modulated the cell cycle progression: decreased the number of cells in S and G2/M cell cycle phase in favor of the increase of the number of cells in G0/G1 cell cycle phase (***p < 0.001). A similar effect was observed for α-ZEL, whereas for β-ZEL such change was not observed.

The modulation of cell cycle progression was also visible in the modulation of cell cycle genes: *CDKN1A*, *CDC2* and *CCNB1* (Fig. 5c). In case of *CDKN1A* a not significant increase in the expression was observed after α-ZEL treatment, with no changes between cell lines as well as LY294002 addition. After treatment with β-ZEL a significant decrease in the expression was observed as compared to control cell lines (0.52 fold, ***p < 0.001) and not significant difference between PC3-FOXO3a- and PC3-CNT cells. In case of control cells treatment with LY294002 resulted in a significant decrease in the expression, whereas for α-ZEL no change was visible and in case of β-ZEL a not significant increase was observed. In case of *CDC2* expression ZELs decreased its expression significantly as compared to control (~ 0.26 fold, ***p < 0.001) and similarly to *CDKN1A* expression, LY294002 decreased the expression, whereas in case of ZELs increased the expression. The difference between PC3-FOXO3a- and PC3-CNT cells was also visible but not significant. A similar expression pattern was observed for another cell cycle modulator *CCNB1*. All the tested treatments resulted in a significant decrease of the expression of *CCNB1* as compared to control respective cell line. The highest decrease in the expression was observed for control treatment with LY294002 (~ 0.4 fold, ***p < 0.001) whereas for ZELs any differences were not observed.

**Figure 6:**
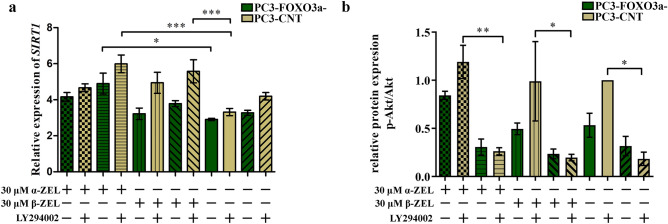
The evaluation of the expression of *SIRT1* and Akt after ZELs treatment. The relative gene expression of *SIRT1* (**a**) and the relative protein expression of p-Akt/Akt (**b**) after ZELs and/or LY294002 treatment. The results are expressed as the mean ± SE. *p* < 0.05 was considered statistically significant, *** p < 0.001, **p < 0.001, *p < 0.05 as compared to the control. α-ZEL- α- zearalenol, β-ZEL- β-zearalenol, *SIRT1*- sirtuin 1.

Subsequently, we decided to evaluate the expression of *SIRT1* and Akt as a main regulators of FOXO3a in cells (Fig. 6a,b). We observed that α-ZEL increased the expression of *SIRT1* as compared to respective control, whereas addition of LY294002 resulted in a higher increase, however not significant. In case of β-ZEL a contradictory effect was observed with a visible difference between PC3-FOXO3a- and PC3-CNT cells. Itself 30 µM of β-ZEL caused almost no change in the expression of *SIRT1* in PC3-FOXO3a- cells, whereas in PC3-CNT cells the increase was observed. Similarly to control cells, addition of LY294002 resulted in a slight, not significant decrease, however a pattern of change of the *SIRT1* expression was sustained. The expression of p-Akt/Akt was evaluated on Western blot and showed that in PC3-CNT cells α-ZEL slightly increased the expression of p-Akt, whereas β-ZEL almost no changed it. As suspected, lack of FOXO3a in cells resulted in a decrease in the expression or all tested mycotoxins as well as control condition, whereas LY29002 decreased it even more.

## Discussion

FOXO3a is considered a potential target for anticancer therapy. This is due to its well-known involvement in the regulation of cell proliferation, transformation, cell apoptosis and autophagy. Its interaction with other major cell signaling pathways also plays an important role: PI3K/Akt, epidermal growth factor receptor (EGFR), mitogen-activated kinase (MAPK) or ERK kinase. Multiple nutrients have previously been reported to modulate FOXO3a in PCa^9^. Most of them interacts with PI3K/Akt signaling and modulates the cell cycle progression, proliferation and induces apoptosis in cells^20^. As showed previous studies that also mycotoxins including DON and ZEN might affect the expression of FOXO3a in PCa cells^17,21^. However none of that studies considerated the active metabolites of ZEN. The present study provides an information of the involvement of both FOXO3a as well as PI3K/Akt signaling pathway in ZELs-induced toxicity in PCa cells. It was also observed that both mycotoxins have different molecular mechanisms induced by ROS in PCa cells, related to the FOXO3a and PI3K/Akt signaling pathway.

The hydroxyl derivatives of ZEN: α-ZEL and β-ZEL are known from estrogenic potency in cells, even higher than ZEN itself in case of α-ZEL^22^, ability to induce oxidative stress^23^ and DNA damage^24^. The justification for undertaking research assessing the impact of ZEN metabolites on cells is the fact that they are detected in human and animal samples^25^. Moreover, the transformation of ZEN to ZELs might happen in liver, lung, kidney as well as prostate^25^. Due to the fact that PTEN loss corresponds with hormone insensitive PCa cells, in this study we used androgen- independent cell line: PC3. Firstly, we confirmed our assumption that ZELs increases the expression of FOXO3a in a significant manner in PCa cells. A similar, dose- dependent effect has been observed previously by Kang et al.^17^ in case of DON in a dose of 2 and 1 µg/mL in porcine IPEC-J2 cells. Sang et al. reported that ZEN in a dose of 20 µM increased the acetyl form of FOXO3a, which serves as the active one in human embryo kidney HEK293 cells^21^. It also seems that the role of FOXO3a might be different in normal prostate cells in which ZEN decreased the expression of *FOXO3a* in a dose-dependent manner^26^. This observation on the other hand was not evaluated for ZEN metabolites as well as in this study we considered the protein level of FOXO3a, not the gene level, which also might be different.

As we have shown, most of the toxic effect of ZEN is associated with induction of oxidative stress, apoptosis and cell cycle modulation in cells^27^. In our present study we observed that α-ZEL is more toxic to β-ZEL as well the mechanism of apoptosis as well as cell cycle arrest is different in PCa cells. First of all we observed that both mycotoxins induced oxidative stress in cells manifested by the increased number of ROS-positive cells. This fact is associated with the previous observation that ZEN itself induces oxidative stress in PCa cells observed in prostate^26^ and liver cells^28^. The observed increase in the number of ROS-positive cells after ZELs treatment was associated with modulation of the expression of SOD1/SOD2 and PARP1 in PC3 cells. ZEN was reported to modulate the expression of *SOD1* in other PCa cells: LNCaP as well DU-145 cells^27^. The decreased expression of *SOD1* was observed in porcine granulosa cells after treatment with ZEN^29^ as well as weanling piglets^30^. The induction of oxidative stress was also observed in HEK293 cells by both ZELs, however in a significantly higher concentration than observed by us: 150 µM and 240 µM of α-ZEL and β-ZEL, respectively^31^. We also observed the decrease in *PARP1* expression suggesting that generation of oxidative stress might be also associated with DNA damage, which was observed previously for other mycotoxin DON^32^. However it was noted that the same dose of α-ZEL or β-ZEL caused a different expression pattern. This fact might be explained by a different susceptibility of cells to mycotoxin- induced toxicity. For both mycotoxins we observed induction of apoptosis in cells, however to higher extend by α-ZEL for which almost no changes were observed in mitochondrial potential as well as caspase 3 and 7 expression. In RAW264.7 macrophages the ROS- induced apoptosis by ZELs was not associated with caspase activation and similarly to our results β-ZEL more significantly induced the mitochondrial depolarization, even when being less toxic to the cells^4^. A different mechanism of cellular death caused by ZELs was also visible in cell cycle analysis. The modulation of cell cycle by ZEN is associated with cell cycle arrest in G2/M cell cycle phase^33^. We also observed a significant arrest in G2/M cell cycle phase in cells treated with α-ZEL, however in β-ZEL treated cells the cell distribution was different indicating cell cycle arrest in G0/G1 cell cycle phase. This observation is consistent with previous one made by Tiemann et al. who observed that 30 µM of β-ZEL increased the number of cells in G0/G1 phase^22^ in porcine endometrial cells. Similar observation for both ZELs was made by Minervini et al. who observed that both ZELs induced an increase in the number of cells in sub-G0 cell cycle phase in granulosa cells^34^.

The modulation of PI3K/Akt signalling pathway by mycotoxins was reported before^7^. It was previously showed that NRF2 activation due to exposure to ZEN is mediated by PI3K/Akt^7^. In macrophages p53, JNK and p38 kinases were activated upon exposure to ZELs^4^, although the authors did not consider involvement of PI3K/Akt pathway in that effect, MAPK kinases has been known to be downstream target of PI3K/Akt signalling cascade^35^. Both ZEN and DON were reported to induce the autophagy and apoptosis of cells via modulation of PI3K/Akt^36^. The involvement of SIRT1 reported to protect cardiac cells against ZELs-induced apoptosis^37^ seems to also supports our hypothesis. The authors suggested that activation of SIRT1 protects the cells against the cytotoxic effect of mycotoxins. In our study we observed that in case of β-ZEL silencing of FOXO3a significantly decreased expression of SIRT1 in the response to the mycotoxin. Previous studies suggested that observed toxicity of ZEN might be associated with epigenetic changes: DNA methylation and histone methylation^38^. SIRT1 is a class III histone deacetylease which regulates tissue homeostasis and deacetylation of histone and not-histone targets in many diseases^39^. The observed modification of the expression of *SIRT1* suggests that ZELs, similarly to ZEN might affect epigenetic modification in cells, as a part of its toxic mechanism. Also we observed that FOXO3a itself only in cell cycle significantly reduced the toxic effect of ZELs in cells. Whereas a simultaneous blockage of FOXO3a as well as PI3K/Akt resulted in a significant decrease in the proliferation of cells and reduction of oxidative stress as well as induction of apoptosis. This results seems to be in line with the work of Das et al. who showed that inhibition of Akt promotes FOXO3a-dependent apoptosis of PCa cells^40^. Based on the fact that also other mycotoxins like DON were reported to induce apoptosis via FOXO3a signalling pathway in different cell line, our results indicate that FOXO3a-PI3K/Akt signalling pathway might be involved in the general cytotoxic effect of mycotoxins, observed over last few years extensively in different cell lines.

## Conclusions

This is the first study which showed that both active metabolites of ZEN induces toxicity in PCa cells and FOXO3a/PI3K/Akt signalling participates in that effect. The results showed that both metabolites possesses a different cytotoxic potential in cells and the observed apoptosis in cells seems to be regulated by a different mechanism, however this statement needs further studies to be confirmed.

## Materials and methods

### Cell culture and ZOLs exposure

Human prostate adenocarcinoma cell line PC3 was purchased from the European Collection of Authenticated Cell Cultures (ECACC) (Sigma Aldrich, Saint Louis, MO, USA). Cells were cultured in RPMI supplemented with 10% heat-inactivated fetal bovine serum (FBS), 2 mM L-glutamine, 1 mM sodium pyruvate, 10 mM 4-(2-hydroxyethyl)-1-piperazineethanesulfonic acid buffer (HEPES) together with 50 U/mL penicillin and 50 μg/mL streptomycin (Thermo Fisher Scientific Inc., Waltham, MA, USA), in a humidified atmosphere of 5% CO2 at 37 °C. Medium without antibiotics and serum was used as experimental.

Stock solutions of α-zearalenol (α-ZEL) and β-zearalenol (β-ZEL) (Sigma-Aldrich, Saint Louis, MO, USA) were prepared in methanol (0.01 M). The final concentrations of α-ZEL and β-ZEL were achieved by adding experimental medium. Cells were treated with ZELs for 24 h. Non-treated cells were used as control.

### Silencing of FOXO3a

Human FOXO3 CRISPR/Cas9 KO plasmids, and control (Scramble) (Scr) were purchased from Applied Biological Materials Inc. (BC, Canada). Three different FOXO3 CRISPR/Cas9 KO plasmids were tested, each encoding the Cas9 nuclease and a target-specific 20 nt guide RNA (gRNA) designed for maximum knockout efficiency: target 1: 5′-CCCGCTCTCTCCGCTCGAAG-3′, target 2 5′-TTTGTCCGGGGAGCTCTCGA-3′ and target 3 5′-CAGAGTGAGCCGT TTGTCCG-3′. CRISPR/Cas9 KO Plasmids and Control CRISPR/Cas9 Plasmid were transfected into PC3 cells using ViralEntry™ Transduction Enhancer (Applied Biological Materials Inc., Canada) according to manufactures' protocol. Briefly, PC3 cells were seeded onto 12-well plate. After 24 h and reaching 60–70% confluency, the culture media were exchanged to experimental medium with plasmid and ViralEntry™ Transduction Enhancer. At 48 h post-transfection, cells from each well were transferred to the next two wells. The next day medium was exchange to 1 mL of selection medium (complete medium containing 2 μg/mL of puromycin). Successful transfection of the FOXO3 and Control plasmids was verified with Western blots. Target 1 was the most efficient and was selected to further experiments.

### Cell viability

The viability of cells was determined with MTT reagent (3-(4,5-Dimethylthiazol-2-yl)-2,5-Diphenyltetrazolium Bromide) (MTT) (Merck Millipore, Burlington, MA, USA). 5 × 10^4^ cells (PC3, PC3-FOXO3a- and PC3-CNT) were seeded on 96-well plates and incubated at standard conditions to reach 90% confluence. Next, the cells were treated with experimental medium containing α-ZEL or β-ZEL (0.001 to 100 µM) for 24 h. PC3-FOXO3a- and PC3-CNT were also treated with 30 µM α-ZEL, 30 µM α-ZEL + LY, 30 µM β-ZEL, 30 µM β-ZEL + LY, LY or medium alone for 24 h. Then four hours prior to the end of the incubation period 5 mg/mL MTT solution was diluted to a final concentration of 0.5 mg/mL in each well and incubated at 37 °C. The formazan crystals formed by viable cells were dissolved in DMSO (100 μL). Absorbance was measured at 570 nm with ELX808IU plate reader (BioTek, Winooski, VT, USA).

### Flow cytometry

PC3-FOXO3a- and PC3-CNT cells (6 × 10^4^ per well) were seeded on 12-well plates and cultured to reach 90% confluence. Then were treated with 30 µM α-ZEL, 30 µM α-ZEL + LY, 30 µM β-ZEL, 30 µM β-ZEL + LY, LY or medium alone for 24 h. Oxidative Stress Assay (Merck Millipore, Burlington, MA, USA) was performed according to the manufacturer’s instructions. The probes were measured on a Muse™ Cell Analyzer (Merck Millipore, Burlington, MA, USA) and standardized against control probes. The analysis was performed in three independent experiments.

PC3-FOXO3a- and PC3-CNT cells apoptosis was determined using the Muse™ Annexin V and Dead Cell Kit (Merck Millipore, Burlington, MA, USA). The cells (6 × 10^4^ /well) were seeded on 12-well plates and left to reach 90% confluence. After exposure to 30 µM α-ZEL, 30 µM α-ZEL + LY, 30 µM β-ZEL, 30 µM β-ZEL + LY, LY or medium alone the cells were detached and suspended in 100 μL of culture medium. The assay was performed according to the manufacturer’s instructions. The analysis was performed in three independent experiments.

Muse™ MitoPotential Assay (Merck Millipore, Burlington, MA, USA) which evaluates the polarization of mitochondria was used to measure the changes in mitochondrial membrane potential (ΔΨm) in PC3-FOXO3a- and PC3-CNT. Simultaneous use of 7-AAD allows evaluation of cell membrane integrity. Cells (approximately 6 × 10^4^ /well) were seeded on 12-well plates and left to reach 90% confluence. Then, the cells were treated with 30 µM α-ZEL, 30 µM α-ZEL + LY, 30 µM β-ZEL, 30 µM β-ZEL + LY, LY or medium alone for 24 h. The assay was performed as recommended by the manufacturer. The probes were standardized against control probes. The experiment was repeated three times.

Propidium iodide (PI) staining in the presence of RNAse was used to evaluate the percentage of cells in the G0/G1, S and G2 phase of cell cycle with the Muse® Cell Cycle Assay Kit (Merck Millipore, Burlington, MA, USA). 3 × 10^5^ /well cells (PC3-FOXO3a- and PC3-CNT) were seeded on 6-well plates and cultured to reach 90% confluence. Next, treated with 30 µM α-ZEL, 30 µM α-ZEL + LY, 30 µM β-ZEL, 30 µM β-ZEL + LY, LY or medium alone for 24 h and trypsinized. The Cell Cycle Assay was conducted according to manufacturer’s recommendations. Cells were analyzed on Muse™ Cell Analyzer (Merck Millipore, Burlington, MA, USA) and compared to control cells. The results were expressed as the percentage of cells in each cell cycle phase. The experiment was conducted in triplicate.

### Real time quantitative polymerase chain reaction (RT-qPCR)

For RNA isolation 4 × 10^5^ PC3-FOXO3a- and PC3-CNT cells were cultured on 60 mm Petri dishes and treated with 30 µM α-ZEL, 30 µM α-ZEL + LY, 30 µM β-ZEL, 30 µM β-ZEL + LY, LY or medium alone for 24 h. Cells were then suspended in TRIzol Reagent (Thermo Fisher Scientific Inc, Waltham, MA, USA) and RNA was isolated according to the manufacturer’s protocol. Isolated RNA was diluted in 50 μL of sterile deionized water was measured with a BioDrop DUO spectrophotometer (BioDrop, Cambridge, UK). 5 μg of total RNA was used to synthesize cDNA using ImProm RT-IITM reverse transcriptase (Promega, Madison, WI, USA) according to the manufacturer’s instructions. RT-qPCR was conducted with LightCycler 96 (Roche, Basel, Switzerland) with 2 μL of cDNA. Primers were designed and verified using Primer-BLAST (National Institutes of Health) (Table 2). The Human Reference RNA (Stratagene, San Diego, CA, USA) was used as a calibrator of reaction. The relative expressions of cyclin B1 (*CCNB1*), cyclin-dependent kinase 1 (*CDC2*), cyclin dependent kinase inhibitor 1A (*CDKN1A*), caspase 3 (*Casp3*), caspase 7 (*Casp7*), poly [ADP-ribose] polymerase 1 (*PARP-1*), protein kinase B (*AKT*), sirtuin 1 (*SIRT1*), superoxide dismutase 1 (*SOD1*), superoxide dismutase 2 (*SOD2*). Ribosomal protein S17 (*RPS17*), ribosomal protein P0 (*RPLP0*), and histone H3.3A (*H3F3A*) were used as a reference genes. The melting curve analysis was performed to confirm the specificity of the product for each primer set. The data was analyzed with the ΔΔCt method. Each reaction was conducted in a duplicate of three repeats of the experiment and expressed as a relative expression.

**Table 2:** Primers used in RT-qPCR.

Gene	Sequence (5′–3′)	Product size (bp)
*CCNB1*	For ACCTATGCTGGTGCCAGTG Rev GGCTTGGAGAGGCAGTA	128
*CDC2*	For TTTTCAGAGCTTTGGGCACT Rev AGGCTTCCTGGTTTCCATTT	100
*CDKN1A*	For GACAGATTTCTACCACTCCAA Rev CTGAGACTAAGGCAGAAGAGT	134
*Casp3*	For GGAATATCCCTGGACAACAGTT Rev TTGCTGCATCGACATCTGT	130
*Casp7*	For GTGGGAACGATGGCAGATGATC Rev AGGGACGGTACAAACGAGGAC	113
*PARP-1*	For TCTTCAAGAGCGATGCCTATT Rev TGAGGTAAGAGATTTCTCGGAA	129
*SIRT1*	For CATAGACACGCTGGAACA Rev GCTTCACAGTCAACTTTGTA	108
*SOD1*	For GCGTGGCCTAGCGAGTTAT Rev ACACCTTCACTGGTCCATTACT	114
*SOD2*	For GGGTTGGCTTGGTTTCAATAAG Rev CTGAAGGTAGTAAGCGTGCTC	136
*RPS17*	For AAGCGCGTGTGCGAGGAGATCG Rev TCGCTTCATCAGATGCGTGACATAACCTG	87
*RPLP0*	For ACGGATTACACCTTCCCACTTGCTAAAAGGTC Rev AGCCACAAAGGCAGATGGATCAGCCAAG	69
*H3F3A*	For AGGACTTTAAAAGATCTGCGCTTCCAGAG Rev ACCAGATAGGCCTCACTTGCCTCCTGC	74

*CCNB1*- cyclin B1; *CDC2*- cyclin-dependent kinase 1; *CDKN1A-* cyclin dependent kinase inhibitor 1A; *Casp3*- caspase 3; *Casp7*- caspase 7; *PARP-1*- poly [ADP-ribose] polymerase 1; *SIRT1*- sirtuin 1; *SOD1*- superoxide dismutase 1; *SOD2*- superoxide dismutase 2; *RPS17*- ribosomal protein S17; *RPLP0*- ribosomal protein P0; *H3F3A*- histone H3.3A.

### Western Blot analysis

For the protein isolation, 8 × 10^5^ PC3-FOXO3a- and PC3-CNT cells were cultured on 100 mm Petri dishes and treated with 30 µM α-ZEL, 30 µM α-ZEL + LY, 30 µM β-ZEL, 30 µM β-ZEL + LY, LY or medium alone for 24 h. The protein isolation and Western blots were conducted as previously described^41^. 30 µg (15 µg for SOD2 (#13141)) of protein samples was used for electrophoresis. Akt (#9272), p-Akt (#4060), FOXO3a (#12829), PARP1 (#46011), SOD1 (#4266) (Cell Signaling Technology, Leiden, WZ, The Netherlands) antibodies were used according to manufacturer’s recommendations. The results were normalized to glyceraldehyde 3-phosphate dehydrogenase (GAPDH) (sc-59540) (Santa Cruz Biotechnology Inc, Dallas, TX, USA) as a reference protein.

### Statistical analysis

Results are expressed as mean ± SE. The one-way ANOVA test was used to calculate statistically significant differences. *p* < 0.05 was considered as statistically significant. GraphPad Prism software (GraphPad Software, La Jolla, CA, USA) was used to carry out all statistical analyses.

### Supplementary Information

Supplementary Information 1.

## Data Availability

The data sets used and/or analyzed during the current study available from the corresponding author on reasonable request.

## References

[1] Reddy KE (2018). Effects of deoxynivalenol- and zearalenone-contaminated feed on the gene expression profiles in the kidneys of piglets. Asian-Austral. J. Anim. Sci..

[2] Zinedine A, Soriano JM, Moltó JC, Mañes J (2007). Review on the toxicity, occurrence, metabolism, detoxification, regulations and intake of zearalenone: An oestrogenic mycotoxin. Food Chem. Toxicol..

[3] Schollenberger M (2006). Natural occurrence of 16 fusarium toxins in grains and feedstuffs of plant origin from Germany. Mycopathologia.

[4] Lu J (2013). Cellular mechanisms of the cytotoxic effects of the zearalenone metabolites α-zearalenol and β-zearalenol on RAW264.7 macrophages. Toxicol. Vitr..

[5] Gajęcka M (2021). Concentration of Zearalenone, Alpha-Zearalenol and Beta-Zearalenol in the Myocardium and the results of isometric analyses of the coronary artery in prepubertal gilts. Toxins.

[6] Grgic D, Varga E, Novak B, Müller A, Marko D (2021). Isoflavones in animals: Metabolism and effects in livestock and occurrence in feed. Toxins (Basel).

[7] Xu W (2022). Role of PI3K/Akt-mediated Nrf2/HO-1 signaling pathway in resveratrol alleviation of zearalenone-induced oxidative stress and apoptosis in TM4 cells. Toxins (Basel).

[8] Kowalska K (2022). Deoxynivalenol induces apoptosis and autophagy in human prostate epithelial cells via PI3K/Akt signaling pathway. Arch. Toxicol..

[9] Habrowska-Górczyńska DE, Kozieł MJ, Kowalska K, Piastowska-Ciesielska AW (2021). FOXO3a and its regulators in prostate cancer. Int. J. Mol. Sci..

[10] Shukla S (2014). Apigenin inhibits prostate cancer progression in TRAMP mice via targeting PI3K/Akt/FoxO pathway. Carcinogenesis.

[11] Imada K (2017). FOXO3a expression regulated by ERK signaling is inversely correlated with Y-box binding protein-1 expression in prostate cancer. Prostate.

[12] Li C, Hu W-L, Lu M-X, Xiao G-F (2019). Resveratrol induces apoptosis of benign prostatic hyperplasia epithelial cell line (BPH-1) through p38 MAPK-FOXO3a pathway. BMC Complement. Altern. Med..

[13] Shukla S, Gupta S (2010). Apigenin: A promising molecule for cancer prevention. Pharm. Res..

[14] Ganapathy S, Chen Q, Singh KP, Shankar S, Srivastava RK (2010). Resveratrol enhances antitumor activity of TRAIL in prostate cancer xenografts through activation of FOXO transcription factor. PLoS One.

[15] Oak C (2018). Diosmetin suppresses human prostate cancer cell proliferation through the induction of apoptosis and cell cycle arrest. Int. J. Oncol..

[16] Shankar S, Ganapathy S, Srivastava RK (2008). Sulforaphane enhances the therapeutic potential of TRAIL in prostate cancer orthotopic model through regulation of apoptosis, metastasis, and angiogenesis. Clin. Cancer Res..

[17] Kang TH, Kang KS, Lee SI (2022). Deoxynivalenol induces apoptosis via FOXO3a-signaling pathway in small-intestinal cells in pig. Toxics.

[18] Yang Y (2018). Transcription factor FOXO3a is a negative regulator of cytotoxicity of Fusarium mycotoxin in GES-1 cells. Toxicol. Sci..

[19] Patane, M. & Shilabye, S. Fusaric acid Fumonisin B1 CO -treatment regulates AMPK signalling and induces Apoptosis in HEPG2 cells. (2019).

[20] Chen Q, Ganapathy S, Singh KP, Shankar S, Srivastava RK (2010). Resveratrol induces growth arrest and apoptosis through activation of FOXO transcription factors in prostate cancer cells. PLoS One.

[21] Sang Y, Li W, Zhang G (2016). The protective effect of resveratrol against cytotoxicity induced by mycotoxin, zearalenone. Food Funct..

[22] Tiemann U, Viergutz T, Jonas L, Schneider F (2003). Influence of the mycotoxins α- and β-zearalenol and deoxynivalenol on the cell cycle of cultured porcine endometrial cells. Reprod. Toxicol..

[23] Agahi F, Álvarez-Ortega N, Font G, Juan-García A, Juan C (2020). Oxidative stress, glutathione, and gene expression as key indicators in SH-SY5Y cells exposed to zearalenone metabolites and beauvericin. Toxicol. Lett..

[24] Fleck SC, Hildebrand AA, Müller E, Pfeiffer E, Metzler M (2012). Genotoxicity and inactivation of catechol metabolites of the mycotoxin zearalenone. Mycotoxin Res..

[25] Ali N, Degen GH (2019). Biomonitoring of zearalenone and its main metabolites in urines of Bangladeshi adults. Food Chem. Toxicol..

[26] Kowalska K (2019). Estrogen receptor β plays a protective role in zearalenone-induced oxidative stress in normal prostate epithelial cells. Ecotoxicol. Environ. Saf..

[27] Kowalska K, Habrowska-Górczyńska DE, Domińska K, Urbanek KA, Piastowska-Ciesielska AW (2020). ERβ and NFκB—Modulators of Zearalenone-induced oxidative stress in human prostate cancer cells. Toxins (Basel).

[28] Rajendran, P. *et al.* Kaempferol inhibits zearalenone-induced oxidative stress and apoptosis via the PI3K/Akt-mediated Nrf2 signaling pathway: In vitro and in vivo studies. *Int. J. Mol. Sci.* **22,** 1–18 (2021). 10.3390/ijms22010217 PMC7794799 33379332

[29] Qin X (2015). Oxidative stress induced by Zearalenone in Porcine granulosa cells and its rescue by curcumin in vitro. PLoS One.

[30] Marin DE, Pistol GC, Neagoe IV, Calin L, Taranu I (2013). Effects of zearalenone on oxidative stress and inflammation in weanling piglets. Food Chem. Toxicol..

[31] Ben Salem, I. *et al.* Crocin protects human embryonic kidney cells (HEK293) from α- and β-Zearalenol-induced ER stress and apoptosis. *Environ. Sci. Pollut. Res.* **23,** 15504–15514 (2016). 10.1007/s11356-016-6741-y 27121014

[32] He Y (2021). Transcriptome analysis of Caco-2 cells upon the exposure of mycotoxin deoxynivalenol and its acetylated derivatives. Toxins.

[33] Ji YM (2022). High-dose zearalenone exposure disturbs G2/M transition during mouse oocyte maturation. Reprod. Toxicol..

[34] Minervini F (2006). Influence of mycotoxin zearalenone and its derivatives (alpha and beta zearalenol) on apoptosis and proliferation of cultured granulosa cells from equine ovaries. Reprod. Biol. Endocrinol..

[35] Shorning BY, Dass MS, Smalley MJ, Pearson HB (2020). The PI3K-AKT-mTOR pathway and prostate cancer: At the crossroads of AR, MAPK, and WNT signaling. Int. J. Mol. Sci..

[36] Gu X (2019). Deoxynivalenol-induced cytotoxicity and apoptosis in IPEC-J2 cells through the activation of autophagy by inhibiting PI3K-AKT-mTOR signaling pathway. ACS Omega.

[37] Ben Salem I (2017). SIRT1 protects cardiac cells against apoptosis induced by zearalenone or its metabolites α- and β-zearalenol through an autophagy-dependent pathway. Toxicol. Appl. Pharmacol..

[38] Karaman EF, Zeybel M, Ozden S (2020). Evaluation of the epigenetic alterations and gene expression levels of HepG2 cells exposed to zearalenone and α-zearalenol. Toxicol. Lett..

[39] Alves-Fernandes DK, Jasiulionis MG (2019). The role of SIRT1 on DNA damage response and epigenetic alterations in cancer. Int. J. Mol. Sci..

[40] Das TP, Suman S, Alatassi H, Ankem MK, Damodaran C (2016). Inhibition of AKT promotes FOXO3a-dependent apoptosis in prostate cancer. Cell Death Dis..

[41] Kowalska K, Habrowska-Górczyńska DE, Domińska K, Piastowska-Ciesielska AW (2017). The dose-dependent effect of zearalenone on mitochondrial metabolism, plasma membrane permeabilization and cell cycle in human prostate cancer cell lines. Chemosphere.

