# Supplementary material for: FOXO3a/PI3K/Akt pathway participates in the ROS- induced apoptosis triggered by α-ZEL and β-ZEL
Source: Sci Rep. 2024 Jun 10;14:13281. doi: 10.1038/s41598-024-64350-8 (PMC11164887; doi:10.1038/s41598-024-64350-8)

Original blots

1, 2- 30 μM α-ZEL; 3, 4- 30 μM α-ZEL + LY294002; 5, 6- 30 μM β-ZEL; 7, 8- 30 μM β-ZEL + LY294002; 9, 10- Cnt; 11, 12- Cnt + LY294002; PC-FOXO3a- (1; 3; 5; 7; 9; 11), PC3-CNT (2; 4; 6; 8; 10 ;12).

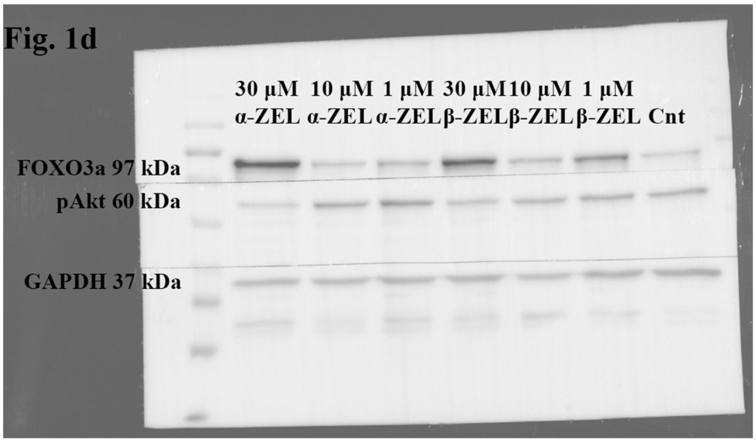

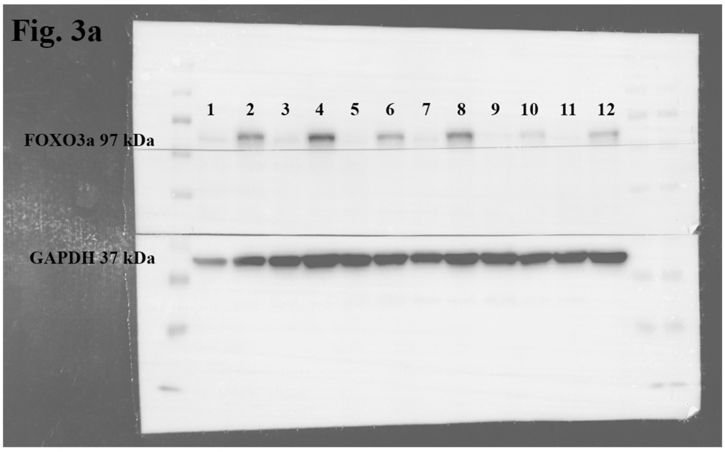

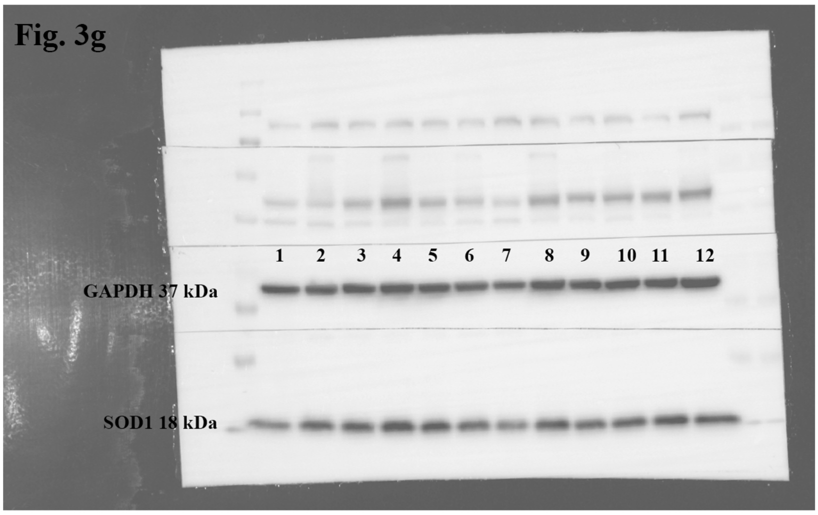

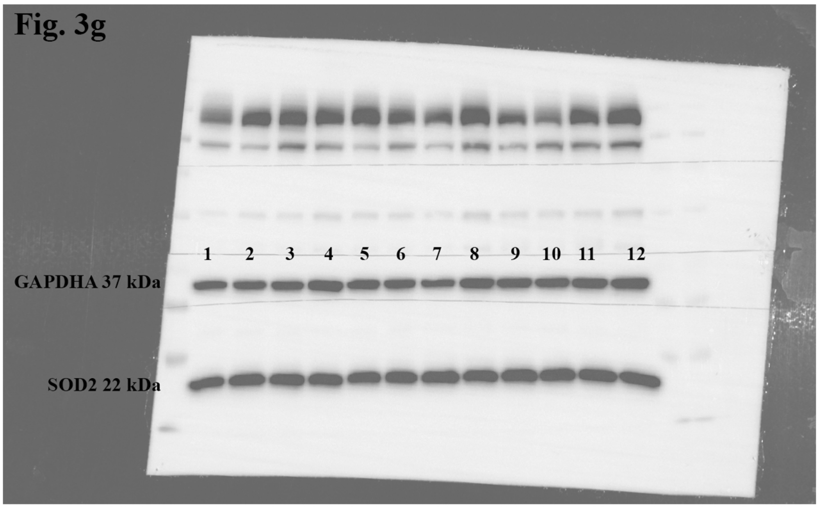

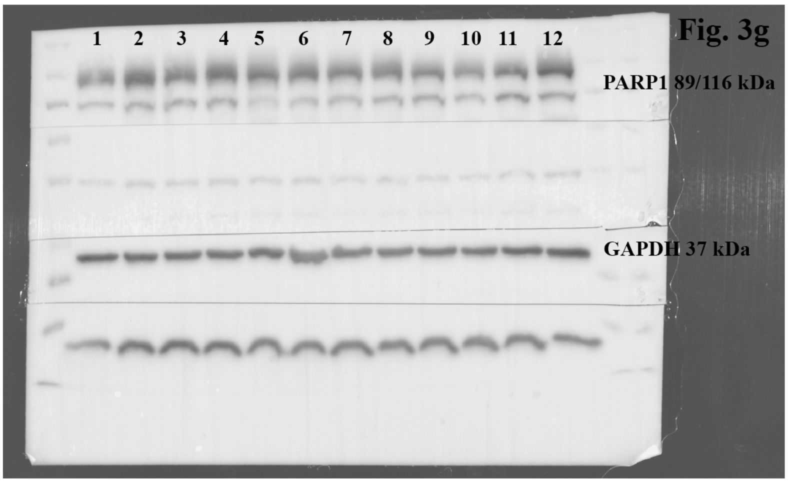

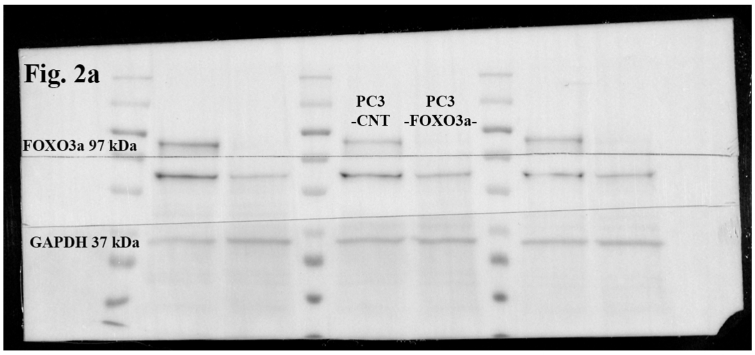

Supplement: Supplementary file 1 — Supplementary Information 1. [file 41598_2024_64350_MOESM1_ESM.pdf]
